# Stevia Rebaudiosides Usage as a Sugar Reduction Tool: A Narrative Review of Their Metabolic, Gut Microbiome and Weight Management Effects in Human Clinical Studies

**DOI:** 10.3390/nu18122002

**Published:** 2026-06-20

**Authors:** Corey Scott, Nikoleta Stamataki, John McLaughlin

**Affiliations:** 1 Cargill, Inc., Wayzata, MN 55391, USA; 2 Division of Diabetes, Endocrinology and Gastroenterology, School of Medical Sciences, Faculty of Biology, Medicine and Health, Manchester Academic Health Sciences Centre, The University of Manchester, Manchester M13 9PT, UK

**Keywords:** stevia, rebaudiosides, sugar reduction, obesity, blood glucose, gut microbiome

## Abstract

**Background/Objectives:** Stevia rebaudiosides represent a class of compounds extracted from the *Stevia rebaudiana* Bertoni plant or produced via yeast fermentation, which provide a sweet taste with little to no calories. These compounds are commercially referred to as stevia and are used in the food industry to reduce sugar in foods and beverages. Stevia is a non-nutritive sweetener (NNS), which is a class of ingredients which represent both artificial and plant-based sweeteners. NNSs are widely used and have been well studied. However, their effects on efficacy for weight management as a sugar reduction tool and overall metabolic effects are inconsistent. Of the approved NNSs for use, stevia is relatively new and one of the least studied. However, recent human clinical research has provided insights into stevia’s metabolic effects, effects on the gut microbiome and effects on weight management when used to replace sugar. The objective of this narrative review of human clinical studies is to provide an overview of the effects of stevia rebaudiosides (largely rebaudioside A) on glucoregulatory and cardiometabolic functions, as well as their effects on gut microbiome and weight management. These studies were typically short term (acute to three months) and heterogeneous by design, and they contained stevia rebaudiosides as lone sweeteners and as part of a binary blend with other NNSs. The majority of metabolic studies on stevia rebaudiosides have evaluated the effects on glucose homeostasis and, to a lesser extent, the effects on cardiometabolic function, the gut microbiome, and weight management. These studies suggest that stevia rebaudiosides have no statistically significant effects on glycemia, insulinemia, blood lipids, appetite hormones, or the gut microbiome. Limited studies suggest that, particularly when compared to sucrose, stevia produces very modest body weight and BMI changes, while studies on subjective appetite and food intake have had inconsistent results. **Conclusions:** Longer-term studies are needed, with more consistent and rigorous design protocols across various populations. However, current human clinical studies suggest that stevia rebaudiosides have a limited impact on metabolic functions, and the observed effects on gut microbiome and changes in body weight, particularly when used to replace sugar, warrant further study.

## 1. Introduction

**Figure 1:**
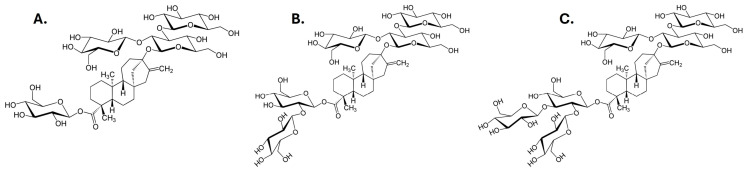
Chemical structures of common stevia rebaudiosides used in commercial food and beverage products: rebaudioside A (**A**), rebaudioside D (**B**), and rebaudioside M (**C**)**.** All stevia rebaudiosides share the same steviol backbone. Rebaudioside A is the major stevia rebaudioside existing in stevia leaves and is tetraglycosylated, whereas rebaudiosides D and M are minor steviol glycosides which are pentaglycosylated and hexaglycosylated, respectively.

Stevia is the general term used for sweet-tasting compounds used as a sweetener which are extracted from the herb *Stevia rebaudiana* (Bertoni), which is a small perennial shrub of the Asteraceae (Compositae) family that is native to South America (Paraguay, Brazil and Argentina). The leaves of this plant, often called “sweet leaf”, have been used by indigenous people for centuries in medicines and to sweeten drinks [1,2]. The sweet taste is a result of the presence of the natural constituents of the plant, called steviol glycosides. Chemically, steviol glycosides are four-ring diterpenes composed of an aglycone backbone called steviol conjugated to various numbers and types of sugar moieties, including glucose, fructose, galactose, rhamnose, xylose, deoxyglucose, and arabinose (Figure 1). More than 60 steviol glycosides have been identified; however, the predominant ones found in *S. rebaudiana* are steviosides (5–10% *w*/*w*) and a subset of steviol glycosides called rebaudiosides: rebaudioside A (reb A) (2–4% *w*/*w*) and reb C (1–2% *w*/*w*) [3].

Steviol glycosides are sweet-tasting compounds roughly 200–400 times sweeter than sucrose on a per-gram basis, but they provide little to no energy [3]. In the United States, extracts from the stevia plant first appeared in 1995 when the Food and Drug Administration (FDA) revised its import alert on stevia leaves and extracts to allow for their use as dietary ingredients in dietary supplements [4]. A high-purity (≥95%) steviol glycoside, rebaudioside A (reb A), received a no-objection letter and GRAS status from the US FDA for use in foods and beverages in 2008 [5], and, to date, the FDA has evaluated more than 50 GRAS notices for the use of various high-purity (95%) steviol glycosides but not whole leaf or crude stevia extracts as sweeteners in food.

Although reb A is the most abundant rebaudioside in the stevia leaf, it is not the sweetest-tasting. Sweeter-tasting and less bitter rebaudiosides (reb D and M) are produced via bioconversion from reb A or fermentation in genetically engineered yeast species of *Saccharomyces cerevisiae* [6] and *Yarrowia lipolytica* [7] that express genes involved in the biosynthesis of steviol glycosides [4]. Steviol glycosides produced via bioconversion and fermentation have undergone toxicological safety evaluations and also received no-objection GRAS notifications from the US FDA [8,9,10]. The benefits of steviol glycosides produced via fermentation or bioconversion, in addition to sweetener-tasting steviol glycosides, include improved sustainability, reduced water use and land use, and lower CO_2_ emissions [3].

In Europe, the European Food Safety Authority (EFSA) approved the use of high-purity steviol glycosides (≥95% rebaudioside A) as a food additive in 2010 [11]. Recently, the EFSA concluded that the inclusion of the 60 steviol glycosides in the proposed specifications for steviol glycoside (E960) would not be of safety concern [12]. In 2023, steviol glycoside production via fermentation was approved by both the EFSA and FDA for use in foods [13].

The Joint FAO/WHO Expert Committee on Food Additives (JECFA) reviewed the safety of steviol glycosides and established an Acceptable Daily Intake (ADI) for steviol glycosides (expressed as steviol equivalents) of 0–4 mg/kg BW/day. Estimates of dietary exposure to stevia-derived ingredients are within JECFA’s established ADI. Renwick et al. [14] estimated reb A intakes for adults, children, and diabetic children using equivalent intake calculations based on existing low- and no-calorie sweetener consumption surveys for North America, Australia, and Europe. This study found that, in the general population, mean intakes among steviol users ranged from 0.4 to 0.7 mg steviol equivalents/kg/d, and for adults and children, high intakes (≥90th percentile) were 1.1–1.7 mg steviol equivalents/kg/d.

Stevia is a non-nutritive sweetener (NNS) which represents a category of ingredients used in foods and beverages to reduce added sugars. Excessive sugar intakes, particularly from sugar-sweetened beverages (SSBs), have been linked to adverse health effects in epidemiological studies, including dental caries, weight gain, diabetes, and cardiovascular disease [15]. Therefore, it is important for reduced or zero-sugar alternatives to help mitigate these disease risks, and as NNSs provide a sweet taste with little to no calories, they are optimal candidates. NNSs have wide uses, appearing in many types of reduced sugar foods and beverages, such as snack foods, yogurt, diet soft drinks, sugar-free desserts, and chewing gum, and also in personal care products, such as toothpaste and mouthwash, as sweeteners. They have been well studied in humans and have global regulatory approval. However, there are concerns surrounding the metabolic effects and efficacy of NNS use for weight management [16]. The intended use of NNSs is to replace sugar, and the human metabolic effects of sugar are well known. Sugars provide sweetness and energy; however, NNSs only provide sweetness. As NNSs uncouple energy and sweetness, the “uncoupling hypothesis” suggests that consumption of NNSs can disrupt normal metabolic processes [17]. Furthermore, many NNSs can travel to the distal gut and interact with gut microflora, and studies suggest that NNSs cause gut dysbiosis, can interfere with the gut microbiome profile and function, and also cause downstream insulin resistance [18]. The scientific literature on the weight management efficacy and metabolic effects of NNS is inconsistent largely due to huge variances in study quality, designs, durations, and populations. Both randomized controlled trials (RCTs) and observational studies are useful in evaluating the effects of NNS and have been performed. NNS RCTs use precise regimens and can control for various factors, and they can help establish causal relationships but often have short- to medium-term evaluations and typically cannot allow for large populations and long durations. Observational studies leverage large populations over several years and can help establish correlations and give indications of long-term effects, but they often cannot control for the under-reporting of data, residual confounding, and reverse causality. In 2023, the World Health Organization Nutrition Guidance Expert Advisory Group (WHO-NUGAG) recommended not using NNSs to manage weight or to reduce the risks of non-communicable diseases (conditional recommendation). The WHO leveraged both randomized controlled trials (RCTs) and observational studies to make their recommendation and acknowledged short-term (up to six months) modest changes in adiposity measures when used to replace sugar (−0.71 kg body weight and −0.14 kg/m^2^ BMI) [19]. The systematic review and meta-analysis also noted limited impact on metabolic effects, including glucose, insulin, blood pressure, and cholesterol from NNS RCTs. However, the recommendation relied more on observational studies, which are often longer term and evaluate larger populations, and these findings suggested adverse health effects on adiposity, type 2 diabetes risks, and cardiovascular disease risks from NNSs and null effects on weight management.

There is a burden to reduce added sugar intake worldwide to help improve weight management and thereby reduce risks of obesity, diabetes, and other related non-communicable diseases, especially for populations such as children and young adults. NNSs may be a useful tool for aiding sugar reduction. Of the approved NNSs with either FDA GRAS status, approved food additive status, or EFSA approval (i.e., saccharin, sucralose, aspartame, acesulfame-K, stevia, neotame, advantame, thaumatin, cyclamate, luo han gou (monk fruit), and aspartame-acesulfame-k salt) [20,21], stevia represents one of the least studied in human clinical studies, and given its increased use as an NNS, knowledge of its metabolic effects and efficacy as a weight management tool through sugar reduction will be useful for consumers, food manufacturers, non-governmental organizations (NGOs), and global regulatory agencies. Historically, the effects of stevia on metabolic outcomes such as glycemia, insulinemia, and cardiovascular disease risks have been studied, but recent human clinical studies have evaluated outcomes, including appetite and energy intakes, effects on the gut microbiome, and weight management effects compared to sugar. Interventions have included various doses of stevia rebaudiosides as a sole sweetener and as part of a blend. Study duration has ranged from acute studies to several months of daily feeding, using comparators such as no intervention, water, or caloric sugars. Diverse populations have been studied, including healthy weight individuals, individuals with overweight/obesity, and individuals with type 1 and type 2 diabetes mellitus or impaired glucose management. The aim of this narrative review is to summarize findings from these studies to better understand the metabolic, gut microbiome, and weight management effects of stevia rebaudiosides alone and against comparators over various time periods and in diverse volunteer groups.

## 2. Methods

A literature search was performed on three electronic databases for this narrative review, which were Cochrane Central Registry of Controlled Trials (Central, which included records from PubMed, Embase, Clinicaltrials.gov, ICTRP and CINAHL), PubMed (which was independently searched), and Scopus, to identify human clinical studies which have evaluated the effects of stevia rebaudiosides on weight management, cardiometabolic effects, glucoregulatory effects, or the gut microbiome as primary or secondary outcomes. A publication on NNS human clinical studies was screened manually and also leveraged [22]. The search period was between 1 January 2000 and 13 March 2026. Search terms included “stevia”, “steviol glycosides”, “stevia rebaudiosides”, “RCT”, “randomized clinical trial”, and “human clinical study.” Filter terms included clinical study, clinical trial, and human, and excluded terms included animal, in vitro, review, and meta-analysis. Publication types searched were limited to journal articles. Full search methods are included in the Supplementary Materials. Inclusion criteria included human studies published in English, with stevia rebaudiosides used as a sweetener alone or as part of a sweetener blend. These studies encompassed various adult populations across BMI ranges (healthy, overweight, and obese) and glucose homeostasis statuses (normal, prediabetes, type 1 diabetes, and type 2 diabetes) and examined effects on weight management (body weight, fat mass, and BMI) and metabolic outcomes (glucoregulatory and cardiovascular disease risks), and the gut microbiome. The scope of these studies includes evaluating stevia rebaudiosides alone or as part of a sweetener blend under non-replacement conditions for metabolic effects and also as a replacement condition (for caloric sugars) for weight management effects. Stevia and other NNSs are often combined with ingredients, such as polyols or other carbohydrates, to increase bulk, improve taste profiles, provide a better mouthfeel, and more closely resemble the granularity of sugar; the addition of these agents provides negligible energy. NNSs are also often combined with other NNSs as sweetener blends, as these combinations are known to have increased sweetening potency over a single NNS in foods and beverages [23]. Stevia has been represented in human clinical studies as a part of a binary sweetener blend, including blending with polyols, other NNSs, and sucrose, and it has been evaluated for effects on post-prandial blood glucose and insulin, cardiovascular disease risks, energy intake, and body weight indices. Stevia rebaudiosides also occur in crude extracts and whole-leaf powders used as food additives or supplements and have been evaluated in human clinical studies. However, as these extracts and powders are not approved for food use by regulatory agencies and are typically non-standardized, highly heterogeneous, and may contain various other components, studies on stevia crude extracts and stevia leaf powders are not included in this review. Stevioside is a steviol glycoside which is the most abundant steviol glycoside in the stevia leaf and has FDA approval for use in food [11]. As stevioside is less sweet and more bitter than stevia rebaudiosides, it is less commonly used in foods and beverages but does appear commercially in supplement form and has also been studied in human clinical trials. Due to the relatively lower use of stevioside in foods and beverages, stevioside human clinical studies are not included in this review. Furthermore, evidence for metabolic effects and weight management from pre-clinical studies (animal, in vitro, cell culture, etc.) is not included in this review.

## 3. Metabolic Effects of Steviol Glycosides

### 3.1. Digestion and Metabolism of Steviol Glycosides

Metabolic studies with steviol glycosides in animals and humans demonstrated that intact steviol glycosides are poorly absorbed after oral exposure, as enzymes and acid present in the upper gastrointestinal tract do not hydrolyze steviol glycosides [24,25]. Reb A has been demonstrated to be degraded in vitro by colonic or fecal bacteria to steviol [26,27]. Steviol rebaudiosides reb B, D, E, and M differ in structure but have all been shown to have the same metabolic endpoint (steviol) as stevioside and reb A when exposed to human fecal bacteria in vitro [28,29]. Gardana et al. demonstrated that the genus *Bacteroidetes* is the one able to degrade steviol glycosides into steviol and that steviol is resistant to further bacterial degradation [26]. There is no current information that suggests that steviol glycosides, although structurally different and varying in sweetening potencies, differ in human metabolism.

In vivo studies in animal models have shown that oral administration of steviol to rats resulted in rapid absorption of steviol into the portal plasma at concentrations of 2–3 µg/mL after two hours [30]. However, after administration of steviol glycosides, steviol detection in portal plasma was much slower, and its presence was sustained over a period of several hours [27]. Nikiforov et al. [24] reported similar metabolism and excretion results for reb A and D in Sprague–Dawley rats. The metabolism of steviol glycosides in humans has been shown to be similar to that of animal models. Steviol glycosides are slowly metabolized by colonic bacteria. Steviol is then absorbed and transported to the liver, where it undergoes conjugation with glucuronic acid to form steviol glucuronide glucuronidation [30]. Peak plasma steviol glucuronide has been demonstrated in humans eight hours after stevioside or 12 h following reb A administration [31]. Reb A and stevioside undergo similar metabolic and elimination pathways in humans, with steviol glucuronide excreted primarily in urine and steviol in feces [32]. There is no evidence that accumulation of steviol glycoside derivatives occurs in the body.

### 3.2. Effects of Stevia Rebaudiosides on Glucose Homeostasis

The intended use of stevia is as a zero-calorie NNS to reduce or replace caloric sugars in the diet, such as sucrose and glucose, which both raise blood glucose levels and elicit an insulin response. As blood glucose and insulin responses outside of the normal range can have adverse effects on health [33], it is important to understand if stevia consumption has any effects on glucose homeostasis. The effects of stevia on glucose homeostasis have been studied in acute post-prandial studies [34,35,36,37,38,39,40,41,42,43,44,45] evaluating blood glucose and insulin and oral glucose tolerance tests (OGTTs). Studies also exist that evaluate longer-term stevia intake effects on fasting blood glucose and insulin levels, as well as post-prandial glucose and insulin responses [43,46,47,48,49,50].

#### 3.2.1. Effects of Stevia Rebaudiosides on Post-Prandial Blood Glucose in Acute Studies

Several studies have assessed post-prandial blood glucose responses following either acute or long(er)-term exposure to stevia alone or blended with another NNS (Table 1). Acute studies have assessed post-prandial blood glucose levels (≤1 day duration) in healthy individuals who have a normal weight [34,35,36,37,38,45,49] or are overweight and obese [37,39,41,48], individuals with type 1 or type 2 diabetes [46,47,49], and women with insulin resistance (IR) [42]. Studies comparing stevia to water as a single agent in a beverage found no statistical differences in post-prandial blood glucose responses [34,35,41]. When comparing the consumption of a stevia-sweetened beverage to a sucrose (or other caloric carbohydrate)-sweetened beverage, the carbohydrate-containing beverages increased blood glucose levels, but stevia beverages/preloads did not [34,35,36,48]. Chupeerach et al. [38] showed a significantly lower glucose iAUC (0–60 min and 0–120 min) following the consumption of a stevia–coconut jelly compared to control coconut jelly in portions yielding 50 g of available carbohydrate. Anton et al. [37] demonstrated a significant reduction in postprandial blood glucose concentrations when stevia was consumed in a midmorning meal compared to sucrose in lean and obese subjects. Tey et al. [36] also showed that, over a 3 h period that included both preload consumption and an ad libitum meal (1 h post-preload consumption), the iAUC for glycemia did not differ between stevia-sweetened or sucrose-sweetened preloads.

#### 3.2.2. Effects of Stevia Rebaudiosides on Post-Prandial Blood Glucose in Longer-Term Studies

Studies were identified to have examined the effects of stevia on postprandial blood glucose or oral glucose tolerance following long(er)-term stevia consumption (>1 day). These studies were conducted on healthy individuals [43,45,46,49,50], individuals with overweight or obesity [41,44,48], individuals with type 1 and type 2 diabetes, or those with impaired fasting blood glucose [46,47,49], and the duration of these interventions varied from two weeks [41] to 3 months [43,44]. From these studies, there was no difference in OGTT or post-prandial blood glucose over time.

#### 3.2.3. Effects of Stevia Rebaudiosides on Post-Prandial and Fasting Blood Glucose as Part of a Sweetener Blend

The effects of stevia on post-prandial blood glucose have been studied as part of sweetener blends which include bulking agents or another NNS. Ng et al. [51] evaluated the effects of a stevia-polyol blend (erythritol and xylitol) against dextrose on glycemic response. The stevia–polyol blend resulted in a significantly lower glucose AUC (0–90 min) compared to a dextrose control. In addition, three RCTs [52,53,54] have shown a significant reduction (with a reduction range of −0.097 mg/dL to −12.5 mg/dL) in postprandial blood glucose compared to sucrose with stevia rebaudiosides or stevia rebaudiosides blended with other NNSs, such as mogroside V or thaumatin, when consumed as a beverage or as part of a reduced-sugar meal in healthy subjects. One of the studies showed an improvement in postprandial blood glucose when combined with an exercise component [54]. In a randomized parallel trial of sixty healthy volunteers, Aswathiah et al. [55] fed a stevia/sucrose blend for 90 days and found no significant differences in blood glucose levels or HbA1C. Shin et al. [56], in a single-arm trial, found no difference in fasting blood glucose following 2 weeks of daily consumption of four sweetener packets, each containing 16 mg of reb A and 986 mg of erythritol, among patients with prediabetes (impaired fasting glucose).

#### 3.2.4. Effects of Stevia Rebaudiosides on Fasting Blood Glucose

Fasting blood glucose levels, typically defined as blood glucose levels after eight hours of no food consumption, serve as a primary measurement for metabolic health assessments and are useful in the diagnosis of diabetes and prediabetes. Therefore, it is important to understand if the use of stevia has any effect on fasting blood glucose levels. Studies using up to 1000 mg/day of reb A [47] with feeding periods up to four months did not significantly influence fasting blood glucose in healthy individuals [45,50], individuals with T1DM [49] or T2DM [47,49], or individuals with impaired fasting blood glucose [42,46]. Villano et al. [48] showed a significant increase in fasting blood glucose after 60 days of daily consumption of a maqui-citrus drink sweetened with stevia (quantity not reported). Two other study arms, involving daily consumption of the same beverage sweetened with sucralose or sucrose, also showed a significant increase in fasting blood glucose. However, the investigators reported that the stevia arm exerted the ‘smallest increase’ compared to the other treatments. Kwok et al. [50] administered 73.6 mg steviol equivalents/day for four weeks and found no significant differences in fasting blood glucose levels in healthy adults. Three other studies showed an improvement in fasting blood glucose following stevia administration [46,47,48,49]. Mayasari et al. [46] examined the effects of daily consumption of a rosella tea sweetened with stevia (5 g of rosella powder and 125 mg of stevia sweetener) twice a day for 14 days in patients with prediabetes (impaired fasting glucose) compared to a no-treatment control group. The investigators observed a significant decrease in fasting blood glucose in the rosella–stevia tea group, but no difference was observed in the control group.

#### 3.2.5. Effects of Stevia Rebaudiosides on Postprandial and Fasting Insulin

In addition to postprandial and fasting blood glucose levels, postprandial and fasting insulin levels are also markers for metabolic health. The effects of stevia rebaudioside supplementation on insulin levels have been evaluated in postprandial studies [36,37,38,39,41,42] and fasting studies [45,46,47,48,49,50] (Table 1). Regarding fasting insulin levels, studies had a duration ranging from 2 weeks [45,46] to 4 months [47] and were conducted in healthy individuals [43,48,49,50], individuals with T2DM [47,49] or T1DM [49], and individuals with impaired fasting glucose (prediabetes) [46]. These studies found that fasting insulin levels were not affected by stevia consumption, with the exception of Suez et al. [45], where stevia caused an increase in non-fasting insulin after 1 week of administration in healthy, normal-weight adults.

Regarding postprandial insulin levels, most identified studies were acute by design (duration < 1 day), and only two studies assessed postprandial insulin after a longer-term consumption period, with Gibbons et al. [41] assessing postprandial insulin levels following a 2-week consumption period of stevia reb M in biscuits and Stamataki et al. [43] evaluating insulin response to an OGTT after 3 months of daily stevia consumption. Studies were performed on healthy individuals with a normal weight [36,37] and individuals with overweight/obesity [39,41,42], and one study included women with insulin resistance [42]. The dosages used varied from 15 mg [42] to 1 g of stevia rebaudiosides [47]. Out of these studies, all showed no significant effects on postprandial insulin responses, while Anton et al. [37] and Gibbons et al. [41] showed significantly lower blood insulin for the stevia condition compared to aspartame or sucrose, respectively.

Blood glucose and insulin levels, both postprandial and fasting, are commonly used as primary predictors for optimal health. Long-term intakes of excessive added sugars (largely from sugar-sweetened beverages) are associated with increased diabetes risks [57]. Glucose and fructose have been shown to elicit a cephalic phase insulin response (CPIR), which is a rise in plasma insulin due to oral sensing of sugars to prepare for incoming glucose [58]. Sweetness in the absence of energy has been shown to elicit a weak CPIR compared to sugars [59,60]. As NNSs reduce or replace sugars, it is important to understand their effects on glucose homeostasis and the factors that influence it. The results from these studies suggest that stevia rebaudiosides do not impact glucose homeostasis and have little to no effect on blood glucose and insulin levels. This includes both fasting levels and postprandial levels in various individuals, including normal weight and overweight individuals and individuals with glucose impairment, such as IR, T1DM, and T2DM. As stevia administration was shown to have no significant effects on postprandial insulin levels in these studies, it is unlikely that stevia elicits a CPIR; however, this requires a specifically designed study. The null effects of stevia itself on glucose homeostasis have been further confirmed in two meta-analyses by Onakpoya & Heneghan [61] on stevia and glycemia, which found no significant effects but suggest a small but significant decrease in fasting blood glucose, whereas Zare et al. [62] found similar lower effects on blood glucose in a dose-dependent fashion (low certainty of evidence) within 1–4 months, particularly in individuals with higher BMI, diabetes, or hypertension, but they observed no effects on HbA1C or insulin levels. Interestingly, two clinical studies [37,41] found lower postprandial blood insulin levels when stevia was incorporated into a food or beverage product compared to sucrose or an artificial sweetener, and this warrants further study. The observation that stevia can improve some measures of glucose homeostasis in some studies is exclusively seen when compared with a sucrose comparator, typically when stevia is used to replace sugars, and these effects are likely relative to the response of sucrose rather than reflecting any physiological or metabolic effects from stevia alone.

### 3.3. Effects of Stevia Rebaudiosides on Gut Hormones and Endocrine Responses

NNSs are used to replace caloric sugars in food and beverages, such as sucrose, which are known to elicit appetite-related hormonal release and influence eating behavior. The effects of NNSs, including stevia rebaudiosides, on gut hormone release are limited and inconsistent with a hypothesis suggesting that NNSs uncouple sweetness and energy and may disrupt normal gut hormone release and function [63]. Very few human studies have evaluated the effects of high-purity stevia rebaudiosides on endocrine hormone release; however, preclinical data do exist and lend some insights. Two studies using ex vivo intestinal models have suggested that reb A induces glucagon-like peptide 1 (GLP-1) and peptide YY (PYY) release. The first study used a porcine ex vivo intestinal model. Tissue segments were collected from various regions of the pig’s small intestine, and reb A in 3 doses (2.5, 12.5, and 25 mM) was shown to stimulate GLP-1 and PYY release [64]. Ripken et al. [65] used an ex vivo model of enteroendocrine cells from the mouse small intestine and demonstrated that reb A augmented the release of incretin GLP-1 (in all three organoid-derived intestinal locations) and PYY (only in the ileum-derived organoid). It was also demonstrated that overnight incubation with reb A increased enteroendocrine cell numbers [65]. On the contrary, Fujita et al. [66] investigated whether secretion of incretins in response to carbohydrates is mediated via taste receptors. The investigators fed Zucker diabetic rats with saccharin, acesulfame-K, D-tryptophan, sucralose, or stevia and showed that none of these sweeteners was shown to augment incretin secretion.

Only a few studies have investigated the effects of stevia consumption on gut hormone response in humans. A study using intraduodenal infusion of 540 mg reb A in 15 healthy lean individuals did not observe any changes in postinfusion levels of CCK, GLP-1, or PYY, and no impact on food intake was observed compared to the placebo infusion (tap water) [67]. In an appetite hormone study, Gibbons et al. [41] measured the release of GLP-1, ghrelin, and pancreatic polypeptide as 2 h iAUC after acute feeding stevia reb M as part of a biscuit, as well as after daily feeding over two weeks. Neither acute nor the two-week feeding of stevia reb M had any effects on the release of the appetite hormones studied. Suez et al. [45] administered stevia reb A for two weeks and reported no significant alterations in GLP-1 levels.

Gut hormones play key roles in appetite regulation and are released in response to sugars. The effects that NNS may have on acute gut hormonal release are not well known, although hypotheses exist suggesting potential disruption of carbohydrate metabolism and impaired hormone release [16,17,18]. As NNSs provide a sweet taste but no energy, the sweet uncoupling hypothesis relates to the gut–brain axis and suggests that NNSs bind to sweet taste receptors throughout the digestive system and may dysregulate gut–brain signaling related to appetite and metabolism through different mechanisms compared to sugars [17,63]. Thus, it is important to understand the effects of NNS on gut hormone release. The administration of stevia rebaudiosides from available studies suggests that stevia has no significant effects on acute appetite hormonal release, including GLP-1, PYY, glucagon, ghrelin, pancreatic polypeptide, and CCK. However, the current studies that have addressed the effects of stevia rebaudiosides on appetite hormones are few, limited, and short-term, and more studies are needed across diverse populations and for longer time periods to accurately evaluate the effects.

### 3.4. Effects of Stevia Rebaudiosides on Markers for Cardiovascular Disease Risks

Cardiovascular disease is multifactorial and is currently the leading cause of death worldwide [68]. High intakes of added sugars and their contributions to excessive caloric intakes are associated with increased cardiovascular disease risks, including high blood pressure and increased blood lipids [69]. The research on NNS and CVD risks is inconsistent. However, the WHO-NUGAG report found no increased CVD risks in human clinical studies, but they did find associations in observational studies [19].

#### 3.4.1. Effects of Stevia Rebaudiosides on Blood Pressure

Stevia rebaudiosides have been evaluated for their effects on blood pressure in healthy normal-weight individuals [43,45,49,50,70,71,72], individuals with overweight/obesity [71], and individuals with T2DM [47,49], and one study included individuals with T1DM [49] (Table 2). The duration of the interventions varied from acute (twenty minutes) [72] to longer term (three to four months) [43,47,49]. In healthy participants, no significant difference in blood pressure was demonstrated following 3 months of 750 mg of stevia rebaudiosides [49], 4 weeks of 1000 mg of reb A supplementation [70], or 3 months of stevia drop use in habitual beverages [43]. However, one study conducted in healthy participants showed a significant increase in blood pressure following one week of stevia use, but no difference was observed when compared to the control (sucrose) condition [71]. In two other studies, blood pressure did not change in patients with T2DM following stevia rebaudioside supplementation [47,49]. However, it should be noted that, in these studies, the participants were advised to continue with their normal medication if they were hypertensive. Kwok et al. [50] administered stevia rebaudiosides at 73.6 mg steviol equivalents/day for 4 weeks and found no significant differences in blood pressure. Similarly, Suez et al. [45] administered stevia reb A at 180 mg/day and found no change in blood pressure compared to the baseline and 2 weeks of exposure. A recent meta-analysis examined the effects of stevia supplementation on blood pressure and reported a significant reduction in systolic blood pressure in favor of stevia rebaudiosides over a placebo but a non-significant reduction in diastolic blood pressure [61].

#### 3.4.2. Effects of Stevia Rebaudiosides on Blood Pressure as Part of a Sweetener Blend

One study evaluated the effects of stevia as part of a binary mixture on blood pressure. In an open-label pilot study, Raghavan et al. [54] administered a mixture of stevia and maltitol for 90 days in overweight and diabetic individuals, and they found no differences in blood pressure.

#### 3.4.3. Effects of Stevia Rebaudiosides on Blood Lipids

Table 3 summarizes the studies carried out on human volunteers that have assessed the effects of stevia rebaudioside consumption on blood lipids. Studies with multiple arms were identified and included participants with T2DM [47,49], healthy participants [49,50,71,73], and overweight/obese individuals [71,73], and one study included participants with T1DM [49]. Stevia rebaudioside doses up to 1000 mg were used, with durations of up to three months. In these studies, total cholesterol, triglycerides, LDL, HDL, and VLDL were not significantly impacted due to stevia rebaudioside consumption.

#### 3.4.4. Effects of Stevia Rebaudiosides on Blood Lipids as Part of a Sweetener Blend

Two studies have evaluated the effects of stevia rebaudiosides as part of a blend on blood lipids. In an acute feeding study, stevia rebaudiosides reb M and reb A were blended with mogroside V and thaumatin, respectively, in beverages with the stevia reb A/thaumatin blend, causing an acute 3% increase in LDL [52]. A stevia/sugar blend significantly decreased total cholesterol, LDL, and VLDL and caused an increase in triglycerides compared to sugar [55].

Taken together, these studies suggest that stevia consumption has little impact on markers for cardiovascular disease risks, including blood pressure and blood lipids, acutely or over several months in various individuals, including those who are normal weight, overweight, or obese and individuals with T1DM and T2DM. However, as CVD risks develop over time, longer-term studies are needed in this area. The effects seen with stevia blends warrant future study.

**Table 2:** Summary of studies assessing the effects of stevia rebaudioside consumption on cardiovascular risk markers.

Reference	Study Type	Volunteers	Duration	Treatments	SBP	DBP	Blood lipids
Stamataki et al. [43]	Randomized, open-label, parallel	28 individuals with normal weight	3 months	10 drops daily of a stevia leaf extract product with habitual drinks	ND	ND	-
Al Dujaili et al. [71]	Randomized, crossover	16 individuals with normal weight, overweight, or obesity	1 week	Stevia (0.2 g) or placebo (5 g of sugar) 3 times a day for 1 week, preferably in a hot drink of their choice	SBP significant increase after stevia ND in the control group (decrease but not significant)	DBP significant increase after stevia ND in the control group	-
Barriocanal et al. [49]	Randomized, double blind, parallel pilot study	16 individuals with T1DM 30 individuals with T2DM 30 individuals with normal weight	3 months	Daily consumption of steviol glycosides 3 × 250 mg capsules (750 mg)	Placebo group exhibited a significant decrease in SBP ND in the stevia group	ND	ND
					ND	ND	ND
					ND	ND	ND
Maki et al. [47]	Randomized, double blind, parallel	122 individuals with T2DM	4 months	Daily consumption of 4 × 250 mg capsules of reb A (1000 mg)	ND	ND	ND
Maki et al. [70]	Randomized, double blind, parallel	100 individuals with normal weight	4 weeks	Daily consumption of 4 × 250 mg capsules of reb A (1000 mg)	ND	ND	-
Zafrilla et al. [73]	Randomized, triple blind, parallel	138 individuals with normal weight or overweight	2 months	Daily consumption of 330 mL maqui-citrus drink sweetened with: Stevia (4 mg/100 mL) Sucralose (4 mg/100 mL) Sucrose (7.5 g/100 mL)	-	-	ND in total cholesterol, HDL, LDL, TG
Kwok et al. 2024 [50]	Randomized, parallel	59 individuals with normal weight	4 weeks	Daily consumption of: 75.6 mg steviol equivalents	ND	ND	No change in total cholesterol, HDL, LDL, VLDL, TG
Suez et al. [45]	Randomized, parallel	120 individuals with normal weight	2 weeks	Daily consumption of 180 mg per day of stevia	ND	ND	-
Rüttgens & Wolf [72]	Randomized 2 × 2 design	72 individuals with normal weight	Acute (20 min)	10 drops of liquid stevia in 300 mL of water or 75 g of glucose	ND	ND	-

Abbreviations: ND denotes no statistical difference detected under the studied conditions; g—grams; DBP—diastolic blood pressure; HDL—high-density lipoprotein; LDL—low-density lipoprotein; mg—milligrams; min—minutes; mL—milliliter; reb A—rebaudioside A; SBP—systolic blood pressure; T1DM—type 1 diabetes mellitus; T2DM—type 2 diabetes mellitus; TG—triglyeride; VLDL—very-low-density lipoprotein. (-) denotes not studied.

### 3.5. Effects of Stevia Rebaudiosides on the Gut Microbiome

The human gut consists of trillions of diverse microorganisms, which are known as the gut microbiome [74]. Often referred to as the second human genome, these serve various functions which are beneficial to the host, such as preventing potentially harmful microorganisms from inhabiting the gut and competing for energy sources. Perhaps the most important role of gut bacteria for the host is the production of short-chain fatty acids (SCFAs), which are produced as a result of indigestible carbohydrate fermentation. SCFAs act as signaling molecules which have multiple roles and beneficial functionalities [75,76,77]. Diet has a profound effect on the gut microbiome profile, as short-term and sustained dietary changes have been shown to alter the gut microbiota [78]. However, the effects of diets and specific dietary components on gut microbiota remain unclear, as there are great inter-individual differences in the responses to dietary components and dietary interventions [79].

Human clinical studies are inconsistent regarding the effects of NNSs on the gut microbiota, as studies have shown that some NNSs perturb the gut microbiota and adversely influence insulin sensitivity [80], while other studies have shown no effects of NNS on the gut microbiome [81,82,83]. Stevia rebaudiosides travel intact to the distal gut and interact with colonic bacteria of the *Bacteroidacea* family, which are shown to eliminate the sugar residues that are conjugated to steviol [28,29]. Thus, it is important to understand any effects that stevia rebaudiosides have on the gut microbiome.

To date, three human clinical studies have evaluated the effects of stevia rebaudiosides on the human gut microbiome (Table 3). In an open-label, single-blind controlled study, Singh et al. [84] used stevia rebaudiosides in the form of liquid drops daily for 12 weeks to evaluate the effects of stevia on the gut microbiome in healthy individuals. Using 16sRNA gene sequencing, the study found no significant changes in alpha and beta diversity due to stevia rebaudiodise intake. In a double-blind, randomised, parallel study, Kwok et al. [50] administered 76.3 mg steviol equivalents/day (representing 25% of the ADI) or 30 g of glucose in a beverage for four weeks in healthy individuals. Changes in the gut microbiome were measured by shotgun metagenomic sequencing, and changes in SCFA production were also measured. There were no measurable changes in the gut microbiome at the phylum, family, genus, or species level, and there were no changes in SCFA production based on time or treatment in this study. Similarly, Suez et al. [45] used shotgun metagenomics sequencing to evaluate the effects of daily 180 mg reb A fed over two weeks on the human gut microbiome in healthy adults in a randomized, double-blind, placebo-controlled study. The study found no significant changes in the gut microbiome when comparing the stevia group to a control group (no NNS) and also to a glucose group.

These three studies are consistent with respect to the effects of stevia on the gut microbiome and did not find stevia rebaudioside administration to significantly impact the diversity, profile, or SCFA production of the human gut microbiome. However, these results, while consistent, should be met with caution as they are limited in number; the study design, measurements, and duration vary; the type of stevia rebaudiosides varies; and the volunteers were healthy adults only. The gut microbiome is known to vary interindividually between persons, and factors such as background diets, age, environment, medicinal and antibiotic use, and health status play a major role in the gut microbiome.

**Table 3:** Summary of studies assessing the effects of stevia consumption on the gut microbiome.

Reference	Study Type	Volunteers	Treatment	Duration	Type of Measurement	Effect
Singh et al. [84]	Randomized, parallel	27 individuals with normal weight	10 drops daily of a stevia leaf extract product with habitual drinks	3 months	16s rRNA gene sequencing	No significant changes in alpha or beta diversity
Suez et al. [45]	Randomized, parallel	120 individuals with normal weight	180 mg reb A	2 weeks	Shotgun metagenomic sequencing	Reported effect on microbiome as stevia/weeks and stevia/days, but not significantly different from glucose and control
Kwok et al. [50]	Randomized, parallel	59 individuals with normal weight	75.6 mg steviol equivalents (reb D and reb M blend)	4 and 8 weeks	Shotgun metagenomic sequencing and SCFA analysis	No change in gut microbiome at family, phylum, genus, or species level or SFCA production due to time or treatment

Abbreviations: 16s rRNA—16 Svedberg unit ribosomal ribonucleic acid; g—milligram; reb A—rebaudioside A; reb D—rebaudioside D; reb M—rebaudioside M; SCFA—short-chain fatty acid.

## 4. Efficacy of Stevia Rebaudiosides for Weight Management Strategies

### 4.1. Effects of Stevia Rebaudiosides on Appetite and Energy Intake

Appetite and energy intake are uniquely connected and play a critical role in both weight management and overall health. Acute appetite can be measured subjectively with visual analog scores such as hunger, desire to eat, prospective food consumption, etc., and energy intake can be measured acutely by ad libitum food intake or longer-term intake with dietary intake records. Appetite dysregulation and excessive energy intake are associated with several metabolic disease risks, including obesity [85]. The intended use of NNSs is to replace sugars, theoretically provide an energy deficit, and aid in weight management. In this context, it is important to understand the effects of stevia rebaudiosides on appetite and energy intake.

#### 4.1.1. Effects of Stevia Rebaudiosides on Short-Term Appetite and Energy Intake

Studies have assessed the effects of stevia rebaudioside consumption on acute (≤1 day) energy intake via ad libitum meals in laboratory settings [34,35,36,37,41,42,67], and they are summarized in Table 4. All of the studies were conducted in individuals with normal weight, with the exception of Gibbons et al. [41], who included both individuals with overweight and obesity. Dosing ranged from 240 ppm to 1000 mg of stevia. Of these studies, two found evidence for significantly lower energy intake associated with stevia consumption compared to either water [34] or sucrose preloads [37]. Tey et al. [36] found no significant difference in overall energy intake throughout the day, as assessed via two laboratory meals (breakfast and lunch) and self-reporting for the rest of the day’s meals. However, taking into account only the preload and the ad libitum meal, energy intake in the stevia condition was 821 kcal (821 kcal from ad libitum meal + 0 kcal from the preload), whereas in the sucrose condition, it was 1002 kcal (742 kcal from ad libitum lunch plus 260 kcal from sucrose preload). This could potentially have led to a statistically significant result if the analysis had been conducted from this perspective. Regarding appetite sensations, out of the studies identified to have examined the acute effects of stevia on appetite ratings, two studies showed improved appetite scores for the stevia preload compared to water on appetite ratings [34,35], and one showed mixed effects [42]. Stamataki et al. [34] found that hunger and desire-to-eat ratings after 30 min were lower for a beverage containing stevia when compared to water. Sambra et al. [42] found greater satiety with stevia at 60 min but a greater desire to eat at 120 min compared to a control. In contrast, Anton et al. [37] showed no difference in appetite ratings between stevia and sucrose preloads, suggesting that caloric and non-caloric sweeteners elicit similar levels of appetite sensations. A study evaluating the direct infusion of reb A into the duodenum did not affect appetite sensations compared to water infusion [67]. Tey et al. [36] reported opposite effects on feelings in appetite, and they found greater suppression of hunger and desire to eat and prospective consumption sensations after the sucrose preload compared to the stevia preload.

#### 4.1.2. Effects of Stevia Rebaudiosides on Short-Term Appetite and Energy Intake with Stevia Rebaudiosides as Part of a Sweetener Blend

One study has evaluated the effects of stevia on appetite as part of a blend. Gibbons et al. [41] found no differences in food intake or appetite ratings between biscuits sweetened with a blend of stevia reb A/thaumatin compared to biscuits sweetened with sucrose.

#### 4.1.3. Effects of Stevia Rebaudiosides on Longer-Term Appetite and Energy Intake

Longer-term studies have assessed energy intake as part of a nutritional intervention with stevia administration, as illustrated in Table 5. Study durations varied from 1 month to 6 months, and the dosage of stevia was up to 1000 mg. Comparators also varied across studies. Higgins & Mattes [44] used sucrose beverages as a control condition, Stamataki et al. [43] compared stevia to no intervention, and in the two studies by Maki et al., stevia was administered in capsules and was compared to control/placebo capsules [47,70]. Two studies showed a significant reduction in energy intake in the stevia group compared to the control group at 3 months of intervention, compared to no intervention [43], or compared to consuming sucrose-sweetened beverages [44]. The rest of the studies showed no significant difference in energy intake following daily stevia administration [47,50,70].

Ultimately, the data on short-term food intake and appetite sensations regarding stevia rebaudiosides is inconsistent, with the majority of studies suggesting no significant effects. A meta-analysis by Rogers et al. [86] showed that, in short-term RCTs (<1 day duration), consumption of an NNS-sweetened preload versus a sucrose-sweetened preload is consistently found to reduce acute energy intake; however, no significant difference was demonstrated in the comparisons between NNSs and water, NNS and unsweetened products, and NNS and no treatment. However, another recent meta-analysis reported significantly lower energy intake during an ad libitum lunch following the consumption of NNS-sweetened preloads compared to unsweetened preloads in the equicaloric comparison (this means NNS preload versus water) [87]. Regarding the equi-sweet comparison and taking the total energy intake into account (preload and meal), there was a trend for reduced energy intake for the NNS-sweetened preload condition, revealing only partial compensation, in line with the results of some of the aforementioned studies on stevia [34,35,37]. The WHO-NUGAG systematic review and meta-analysis on NNS reported a 569 kJ/day reduction in energy intake and 38 g/day reduction in sugar intake when replacing sugar with NNS [19]. The results from recent meta-analyses conclude that the substitution of caloric sugars with NNS in sustained studies leads to a reduction in energy intake [87,88]. However, a specific study on stevia found no effect on energy intake [89]. No significant effects on energy intake were demonstrated when NNSs were compared with water or no intervention, and inconsistent findings were observed when NNS was delivered in capsules versus placebo capsules. These observations led the investigators to conclude that the effects of NNSs on energy intake are most likely due to their lack of energy content as opposed to their taste or any post-absorptive effects. However, more studies are required in the comparison of stevia vs. no treatment or water, given the effects on energy intake shown in recent RCTs [34,43].

### 4.2. Effects of Stevia Rebaudiosides on Weight Management Effects: Body Weight, BMI, and Fat Mass

The relation between NNS intake and body weight has been examined by several epidemiological studies and RCTs, with RCTs providing the most reliable evidence regarding cause-and-effect relationships. In epidemiological studies, NNSs are often analyzed as a group, whereas in RCTs, individual NNSs can be evaluated. Data from meta-analyses of cohort studies are conflicting and have shown either no [84] or a positive association [88,90] between NNS use and changes in BMI or body weight. Nevertheless, causal associations cannot be claimed with data from observational studies, and these data are difficult to interpret, as associations might be due to misreporting of dietary intakes, confounding, or reverse causality. In addition, NNSs are diverse molecules and differ widely in their chemical structure, absorption, metabolism, and excretion, making conclusions of their effects on weight management as a category challenging. In contrast to the conclusions from prospective cohort studies, findings from RCTs on individual NNS typically suggest some benefits for weight management. Meta-analyses of RCTs comparing NNS vs. sugars, water, or no comparator showed a beneficial effect of NNS compared with caloric sugars on body weight, whereas comparisons to water or no comparator did not show significant or consistent results [86,88].

The effects of stevia consumption on weight management have been evaluated in RCTs with endpoints, including body weight, BMI and fat mass, and they are summarized in Table 6. The interventions ranged in duration from 1 week to 2 years; the health status of the participants included healthy individuals [43,44,49,50,70,71,91] and individuals with T2DM [47,49] and T1DM [49], and the BMI ranged from normal to obese. Stevia doses ranged from 13.2 mg/day to 1000 mg/day and were used in the form of capsules or incorporated into a beverage. Four studies (including some groups) found no effects of stevia administration on body weight indices, including body weight, BMI, and fat mass, compared to a placebo, although the primary objective of these studies was not weight management [47,48,49,70]. One study comparing stevia, sucralose, and sucrose consumption for 2 months found that both the sucrose and stevia groups lost fat mass [48]. Similar results were obtained in the study by Al-Dujaili et al. [71], who demonstrated a non-significant reduction in body weight following 7-day supplementation with stevia as opposed to the control condition (sugar consumption), which led to a non-significant increase in body weight. A twelve-week study specifically designed to evaluate the effects of several NNSs (including stevia) on body weight found benefits of NNS. Higgins & Mattes [44] conducted a five-parallel-arm RCT to compare the effects of consumption of four commonly consumed NNS (aspartame, saccharin, reb A, and sucralose) and sucrose in beverages on body weight and body composition over 12 weeks in adults with overweight or obesity. The results showed that consumption of reb A did not change body weight significantly from baseline. However, sucrose led to increased body weight across the 12 wk intervention, and the difference in body weight for the reb A group was significantly lower than the sucrose group at weeks 4, 6, 8, and 12 (*p* < 0.05).

The difference in body weight between the stevia reb A group and sucrose was −1.37 kg (*p* = 0.008), and gynoid fat mass was significantly lower in all NNS groups compared to sucrose (*p* < 0.001). Stamataki et al. [43] administered stevia daily for three months via stevia drops in healthy individuals’ habitual beverages, and they showed the main effect of stevia on body weight change (*p* = 0.026): The control group significantly increased their body weight by +0.89 kg as opposed to the stevia group, which demonstrated a decrease in body weight of −0.22 kg. Kwok et al. administered [50] 73.6 mg of stevia in beverages or 30 g of sucrose to healthy individuals for 4 weeks and found no difference in weight, but they did observe a −0.3 kg/m^2^ difference in BMI between the stevia and sucrose group. In a small six-week study, Daher et al. [91] administered 0.375 mg/kg of stevia, 0.507 mg/kg of sucralose or no NNSs in normal-weight healthy individuals, and they reported a small but statistically significant difference in weight in the sucralose and no-NNS groups only. The study found no difference in BMI.

#### Effects of Stevia Rebaudiosides on Appetite, Weight, BMI, and Fat Mass with Stevia as Part of a Sweetener Blend

Stevia sweetener blends have been evaluated for their effects on appetite and weight management. A study using a blend of stevia reb M with mogroside V and stevia reb A with thaumatin had no acute effect on energy intake compared to sugar [52]. An open-label, single-arm pilot study carried out on overweight and prediabetic individuals consuming a blend of stevia and maltitol found small reductions in both weight and BMI (−1.6 kg and −1.3 kg; −0.67 kg/m^2^ and −0.52 kg/m^2^; *p* < 0.05), respectively, in overweight and prediabetic individuals [54]. A 90-day study on free-living, slightly overweight individuals comparing an additional 5 g of sugar or adding 2.5 g/day of a stevia sugar blend to their diet found a significant weight reduction difference of −1.496 kg (*p* < 0.0001) in the stevia sugar blend group compared to the sugar group [55].

Overall, the studies on the effects of stevia on weight management are very limited and inconsistent, and they cannot properly address weight management largely due to the study design, i.e., short intervention durations, low number of volunteers, lack of statistical power, and weight management not being a primary objective in some of the studies. Studies designed to measure body weight and BMI changes specifically, and one designed to evaluate the gut microbiome, did report small changes in body weight and BMI for the stevia group alone and also when compared to a caloric sugar [43,44,50]. Studies on fat mass are inconclusive. As the main use of NNSs is to reduce or replace sugars, it is important to include the correct comparator, and recent studies [92,93] and meta-analyses [88] comparing NNS with sugar suggest greater weight loss when compared with sugar than compared with water. Regarding stevia specifically, more longer term and specifically designed weight management studies with comprehensive measures and proper controls are needed in order to inform future systematic reviews and meta-analyses on weight management efficacy. Short-term studies evaluating appetite and energy intake are conflicting, with a recent meta-analysis [89] of eleven clinical studies finding no significant changes in any appetite scores following stevia consumption (very low to moderate certainty with respect to the evidence). This systematic review advocated for more long-term clinical trials to obtain more accurate results, as well as more studies on energy intake, as the current literature is insufficient to allow for meta-analysis.

NNS blends are very useful for improvements in sensory and functional properties of foods and beverages; however, they are not well studied in human clinical trials. Presently, the studies evaluated in this review fail to suggest material differences in stevia blends compared to stevia as a single NNS, and more research on stevia blends is warranted in this area.

## 5. Limitations

The narrative review focuses on human clinical studies using stevia rebaudiosides, which evaluated certain metabolic effects (glucoregulatory and cardiometabolic), effects on the gut microbiome, and effects on weight management. This was not a systematic review, and there was no meta-analysis performed. As such, no effect size estimation, study quality assessment, and outcome-level certainty of evidence assessment were performed, which represents a limitation. However, systematic reviews and meta-analyses exist regarding stevia, glycemia, and HbA1c [62] and appetite [89]. Our search strategy included English-language-only studies, and although our search did find three non-English studies, which we did not include, we cannot discount the likelihood of relevant studies in a non-English language which were not evaluated. In addition, another limitation is likely publication bias, given the abundance of small, short-term studies and also the likelihood of unpublished studies not included. This review covers a diverse population; however, the findings on metabolic effects and weight management found in these studies may not be generalizable, as there are few studies on relevant populations, such as the elderly, children, high-frequency users, and persons with chronic diseases, or the effects of different food and beverage matrices. This narrative review contains studies evaluating stevia as part of a blend [51,52,53,54,55,56]. Because NNSs differ widely in their structure, metabolism, and excretion, the effects observed in blend studies cannot therefore be solely attributed to stevia rebaudiosides. Many human clinical studies using stevia leaf powders and crude liquid extracts exist, and they include stevia rebaudiosides as a minor component, as well as steviosides. Steviosides have been evaluated in human clinical studies, albeit at a much lower frequency than rebaudiosides. Stevioside was not included in this review, which represents a limitation. Historically, potential uses of stevioside, stevia powders, and stevia leaf extracts for medicinal, therapeutic, and oral care purposes as a supplement appear in the scientific literature, as reviewed in [94,95]. Although many of these studies suggest some benefits of stevia, several other components in leaf powder and crude extracts, which can contribute to these benefits and these types of studies, fell out of the scope of this review.

## 6. New Research Needs

A significant number of human clinical studies have been performed on stevia rebaudiosides regarding glucose management, cardiovascular disease risks, appetite, energy intake, and weight management. Currently, these studies are mostly short-term, and although they provide some insights, they cannot support claims about longer-term metabolic effects, high-frequency real-world use, or effects in understudied populations. Stevia rebaudiosides, as a lone sweetener or as part of a blend, have been studied in various human populations, and new populations, including children, the elderly, high-frequency users, and individuals with chronic diseases, also warrant study, as sugar reduction and weight management are relevant in these populations. Although current studies suggest no impact on metabolic effects of stevia, which have been confirmed in some systematic reviews and meta-analyses, these findings will further benefit from longer-term studies over several years, dose–response studies, and comparator studies. As the blending of stevia rebaudiosides with other NNSs improves sensory qualities, it is also important to study stevia rebaudioside blends further. In contrast to many metabolic studies, there are relatively few gut microbiome and weight management studies on stevia rebaudiosides, and more representative research is needed in this area. Ultimately, to fully understand the effects of stevia rebaudiosides on weight management, including appetite, food intake, and weight gain/loss, there is a need for high-quality studies with precise standardized methods [96]. These studies can then inform future systematic reviews and meta-analyses to better understand effect sizes and certainty of evidence.

## 7. Conclusions

Stevia rebaudiosides are plant-based NNSs that are increasing in use but have been studied much less in human clinical studies relative to other NNSs. NNS use is inconsistent regarding metabolic effects, effects on the gut microbiome, and its efficacy towards weight management despite global regulatory approval. To date, RCTs evaluating the metabolic and gut microbiome effects of stevia rebaudiosides suggest no significant impacts, with the latter represented by very limited and emerging studies. The data on stevia rebaudiosides for weight management are few and inconsistent, with studies suggesting modest changes in body weight when used to replace sugar. As both weight management and sugar reduction are becoming major global health priorities for consumer packaged goods companies, regulatory agencies, and consumers, the need for sugar reduction tools is urgent. The findings from the human clinical RCTs in this narrative review suggest that stevia rebaudiosides can be used as a tool to help reduce added sugars, as they do not significantly impact metabolic functions and may result in modest weight change in the short term when replacing sugar.

## Figures and Tables

**Table 1:** Human studies evaluating the effects of stevia on blood glucose and insulin levels.

	Postprandial Blood Glucose and Insulin					
	Acute Studies (≤1 day)					
Reference	Study Type	Volunteers	Treatments		Glucose	Insulin
Stamataki et al. [34]	Randomized, double blind crossover	20 individuals with normal weight	Beverage with 240 ppm reb A		ND vs. water, decrease vs. glucose, sucrose, and maltodextrin	-
Farhat et al. [35]	Randomized, single blind crossover	30 individuals with normal weight	Beverage with 1000 mg reb A		ND vs. water, decrease vs. sucrose	-
Chupeerach et al. [38]	Crossover	12 individuals with normal weight	Listed as coconut jelly containing reb A		Decrease in iAUC (0–60 min and 0–120 min) for stevia vs. control product (coconut jelly)	ND in insulin iAUC (0–30 min, 0–60 min, 0–90 min, 0–120 min)
Tey et al. [36]	Randomized, double blind crossover	30 individuals with normal weight	Beverage with 0.33 g reb A		ND in AUC (3 h beverage + meal) vs. sucrose or other NNSs During the first hour (only beverage), decreased response for stevia vs. sucrose	Preload only: Sucrose group-significant increase in iAUC vs. other groups (3 h, meal included): ND across treatments
Tey et al. [40]	Randomized, double blind crossover	30 individuals with normal weight	Beverage with 0.33 g reb A		ND in 24 h glycemic profiles vs. sucrose or other NNS	-
Anton et al. [37]	Single-blind, crossover	31 individuals with normal weight or obesity	Listed as tea and crackers with cream cheese sweetened with stevia (Whole Foods 365 brand)		AUC glucose was lower for stevia compared to aspartame and sucrose	AUC insulin was lower for stevia compared to aspartame and sucrose
Samakkarnthai et al. [39]	Randomized crossover	20 individuals with normal weight or obesity	200 mg stevia listed as pure extracted steviol glycosides		ND AUC for glucose (0–120 min)	ND AUC for insulin (0–120 min)
Gibbons et al. [41]	Randomized double blind crossover	53 individuals with normal weight or obesity	Biscuits with fruit filling containing 21 g of sucrose, 18 mg of stevia reb M, or 2.8 mg of Neotame		2 h iAUC was lower compared to sucrose	2 h iAUC was lower compared to sucrose
Sambra et al. [42]	Randomized double blind crossover	33 individuals (Women) with insulin resistance	Beverage with 15 mg reb A		Serum glucose was higher at 30 min and 60 min after OGTT compared to tagatose and water	No insulin measure; C-peptide iAUC was higher for stevia than water or tagatose
	Longer-Term Studies (>1 day)					
Reference	Study Type	Volunteers	Type of Stevia	Duration	Glucose	Insulin
Stamataki et al. [43]	Open-label, parallel	28 individuals with normal weight	Stevia drops (from extract)	3 months	ND in OGTT	ND in insulin response to an OGTT before or after the intervention or between groups
Higgins & Mattes [44]	Randomized, double blind, parallel	154 individuals with overweight or obesity	1.25–1.75 L of colored, fruit-flavored beverage sweetened with reb A	3 months	ND in OGTT	-
Gibbons et al. [41]	Randomized double blind crossover	53 individuals with overweight or obesity	Biscuits with fruit filling containing 21 g of sucrose, 18 mg of stevia reb M or 2.8 mg of Neotame	2 weeks	ND in 2 h post-prandial iAUC glucose	2 h post-prandial iAUC was lower compared to sucrose
Villano et al. [48]	Randomized, triple blind, parallel	136 individuals with overweight	NR (in a maqui-citrus drink)	2 months	Increase in fasting BG in all arms (stevia showed ‘smallest increase’ vs. sucrose and sucralose	ND
Mayasari et al. [46]	Listed as quasi-experimental	24 individuals with impaired fasting blood glucose	Rosella tea with 125 mg stevia	2 weeks	Decrease in fasting BG in the rosella-stevia group (ND in control)	-
Barriocanal et al. [49]	Randomized, double blind, parallel	30 healthy individuals 16 individuals with T1DM 30 individuals with T2DM	Daily consumption of: steviol glycosides 3 × 250 mg capsules (750 mg)	3 months	ND (increase in T1DM placebo group vs. baseline)	ND
Maki et al. [47]	Randomized, double blind, parallel	122 individuals with T2DM	Daily consumption of 4 × 250 mg capsules of reb A (1000 mg)	4 months	ND	ND
Suez et al. [45]	Randomized parallel	120 individuals with a healthy weight	180 mg reb A	2 weeks	ND in OGTT	Increase in non-fasting insulin after 1 week of administration
Kwok et al. [50]	Randomized parallel	59 individuals with a healthy weight	75.6 mg steviol equivalents (reb M/reb D)	4 weeks	ND in fasting blood glucose	ND

Abbreviations: ND denotes no statistical difference detected under the studied conditions; AUC—area under curve; BG—blood glucose; h—hour; iAUC—incremental area under curve; mg—milligrams; OGTT—oral glucose tolerance test; min—minutes; reb A—rebaudioside A; reb M—rebaudioside M; reb D—rebaudioside D; T1DM—type 1 diabetes mellitus; T2DM—type 2 diabetes mellitus. (-) denotes not studied.

**Table 4:** Human studies examining the acute effects of stevia on appetite and food intake.

Reference	Study Type	Volunteers	Treatments	Appetite	Energy Intake
Stamataki et al. [34]	Randomized, double-blind crossover	20 Individuals with normal weight	Fixed breakfast followed by a 3–4 h fast and then consumption of the following preloads: 330 mL water 330 mL water + 240 ppm stevia 330 mL water + 12% glucose 330 mL water + 12% sucrose 330 mL water + 12% maltodextrin 30 min later, ad libitum meal	Hunger AUC (0–30 min), prospective consumption (0–30 min), and desire to eat (0–60 min) were significantly lower in the stevia, glucose, and sucrose conditions vs. water. Fullness (0–30 min) sign is higher vs. water.	Total energy intake (meal + preload) is lower for stevia vs. water (energy difference: 105 kcal).
Farhat et al. [35]	Single-blind crossover	30 Individuals with normal weight	Fixed breakfast followed by a 3 h fast and then the following preloads: 300 mL water 300 mL water + 60 g sucrose 300 mL water + 1 g stevia (type not mentioned) 30 min later, ad libitum pizza intake	Hunger and desire-to-eat ratings were significantly lower for stevia and sucrose vs. water.	No difference in energy intake across conditions, either at ad libitum meal or from whole-day food diaries.
Tey et al. [36]	Randomized, double-blind crossover	30 Individuals with normal weight	Equi-sweet beverages at 11 am after standardized breakfast: 0.44 g aspartame in water 0.63 g monk fruit in water 0.33 g stevia (reb A) water 65 g sucrose in water 12 pm: ad libitum lunch monitoring of postprandial responses for the subsequent 2 h	Desire to eat, hunger, and prospective consumption ratings from 30 to 60 min were significantly higher for the NNS compared to sucrose (and fullness significantly lower).	Meal-only energy intake was lower for sucrose compared to NNS. Compensation for the ‘missed calories’ was 73% for stevia (107% for aspartame, 93% for monk-fruit). Total EI (whole day, self-reported) did not differ across conditions.
van Avesaat et al. [67]	Randomized, double-blind crossover	15 Individuals with normal weight	After an overnight fast and 150 min after the ingestion of a standardized liquid meal (162 kcal): 120 mL water 540 mg reb A in 120 mL water (Intraduodenal infusion) 15 min after the infusions, participants were offered an ad libitum lunch meal	No significant differences were observed in hunger, desire-to-eat, and satiety ratings between reb A and water infusions.	No difference in energy intake in the ad libitum meal between reb A and water infusions.
Anton et al. [37]	Single-blind crossover	31 Individuals with normal weight and obesity	400 g preload of tea and crackers with cream cheese sweetened with stevia or aspartame or sucrose (amounts not specified) 20 min prior to ad libitum lunch Also, 20 min prior to dinner, the test preloads were consumed. Total daily energy intake assessed (in the laboratory)	Hunger and satiety levels did not differ by condition at any time point.	Significantly lower total daily energy intake in the stevia and aspartame conditions compared to sucrose (mean difference between stevia and sucrose: 300 kcal).
Gibbons et al. [41]	Randomized, double-blind crossover	53 Individuals with overweight and obesity	Biscuits with fruit filling containing 21 g sucrose, 18 mg stevia reb M, or 2.8 mg Neotame	Appetite sensations were similar for all treatments.	Energy intake was similar for all treatments.
Sambra et al. [42]	Randomized, double-blind crossover	33 Individuals (women) with insulin resistance	15.3 mg reb A before a 75 g glucose OGTT compared to water (control) and tagatose	Greater satiety for stevia and D-tagatose vs. control at 60 min and greater desire to eat for stevia vs. control at 120 min.	-

Abbreviations: g—grams; mg—milligrams; min—minutes; mL—milliliter; OGTT—oral glucose tolerance test; reb A—rebaudioside A; reb M—rebaudioside M; kcal—kilocalories; NNS—nonnutritive sweeteners. (-) Denotes not studied.

**Table 5:** Summary of human studies assessing energy intake following longer-term stevia consumption.

Reference	Study Type	Volunteers	Treatment	Duration	Energy Intake
Stamataki et al. [43]	Randomized, open-label, parallel	28 individuals with normal weight	10 drops daily of a stevia leaf extract product with habitual drinks	3 months	The main effect of group on energy intake (difference from baseline): the stevia group showed a significant difference. Decrease in energy intake at 3 months vs. baseline.
Higgins & Mattes [44]	Randomized, single-blinded parallel comparator NNS to sucrose	154 individuals with overweight or obesity	1.25–1.75 L of colored, fruit-flavoured beverage sweetened with reb A	3 months	Mean energy intake for the sucrose group was significantly increased vs. stevia. At week 12, energy intake was significantly increased for sucrose vs. stevia.
Maki et al. [47]	Randomized, double blind, parallel	122 individuals with T2DM	Daily consumption of: 4 × 250 mg capsules of reb A (1000 mg)	4 months	ND
Maki et al. [70]	Randomized, double blind, parallel	100 individuals with normal weight	Daily consumption of: 4 × 250 mg capsules of reb A (1000 mg)	1 month	ND
Kwok et al. [50]	Randomized, parallel comparator NNS to sugar	59 individuals with normal weight	75.6 mg steviol equivalents (reb D/reb M)	4 weeks	ND, but reduced sugar and carbohydrate intake in the stevia group

Abbreviations: ND denotes no statistical difference detected under the studied conditions. mg—Milligrams; reb A—rebaudioside A; reb D—rebaudioside D; reb M—rebaudioside M.

**Table 6:** Summary of studies assessing the effects of long-term stevia consumption on body weight indices.

Reference	Study Type	Volunteers	Duration	Treatments	Body Weight	BMI	Fat Mass
Villano et al. [48]	Randomized, triple blind, parallel	136 individuals with overweight	2 months	Daily consumption of 330 mL maqui-citrus drink sweetened with: Stevia (4 mg/100 mL) Sucralose (4 mg/100 mL) Sucrose (7.5 g/100 mL)	-	ND	ND between groups (both sucrose and stevia decreased fat mass)
Stamataki et al. [43]	Open-label, parallel	28 individuals with normal weight	3 months	10 drops daily of a stevia leaf extract product with habitual drinks Control: no intervention	Main effect of group on body weight change (control increased weight)	Main effect of group on BMI change from baseline	-
Higgins & Mattes [44]	Randomized, single blind, parallel NNS comparator to sucrose	154 individuals with overweight or obesity	3 months	1.25–1.75 L beverage sweetened with reb A or sucrose (Volume depended on baseline participant’s weight)	Increase in weight for sucrose is significantly higher than stevia at weeks 4, 6, 8, and 12	Increase in BMI in the sucrose group; no difference in the stevia group	Total fat mass (not controlling for baseline body weight) increased significantly in the sucrose group
Al-Dujaili et al. [71]	Randomized crossover	16 individuals with normal weight, overweight or obesity	1 week	Stevia (0.2 g) or placebo (5 g of sugar) 3 times a day for 1 week, preferably in a hot drink of their choice	ND	-	-
Barriocanal et al. [49]	Randomized, double blind, parallel	16 individuals with T1DM	3 months	Daily consumption of: steviol glycosides 3 × 250 mg capsules (750 mg) Placebo capsules	ND	ND	-
	Randomized, double blind, parallel	30 individuals with T2DM			ND	ND	-
	Randomized, double blind, parallel	30 individuals with normal weight			ND	ND	-
Maki et al. [47]	Randomized, double blind, parallel	122 individuals with T2DM	4 months	Daily consumption of: 4 × 250 mg capsules of reb A (1000 mg)	ND	ND	-
Maki et al. [70]	Randomized, double blind, parallel	100 individuals with normal weight	4 weeks	Daily consumption of: 4 × 250 mg capsules of reb A (1000 mg)	ND	ND	-
Daher et al. [91]	Randomized, parallel	20 individuals with normal weight	6 weeks	0.375 mg/kg stevia 0.507 mg/kg sucralose No NNS	Small but statistically significant weight loss for the sucralose and control groups only	ND	-
Kwok et al. [50]	Randomized, parallel comparator to sucrose	59 individuals with normal weight	4 weeks	4-week daily consumption of 75.6 mg steviol equivalents (reb D/reb M) 4-week daily consumption of 30 g glucose	ND	−0.3 kg/m^2^ BMI reduction in the stevia group compared to the sucrose group	-

Abbreviations: ND denotes no statistical difference detected under the studied conditions; BMI—body mass index; g—grams; kg—kilogram; L—liter; mg—milligrams; mL—milliliter; reb A—rebaudioside A; reb D—rebaudioside D; reb M—rebaudioside M; T1DM—type 1 diabetes mellitus; T2DM—type 2 diabetes mellitus. (-) denotes not studied.

## Data Availability

No new data were created or analyzed in this study. Data sharing is not applicable to this article.

## Supplementary Materials

The following supporting information can be downloaded at https://www.mdpi.com/article/10.3390/nu18122002/s1, Search results are from Cochrane Central, PubMed, and Scopus.

## Abbreviations

The following abbreviations are used in this manuscript:

ADI	Acceptable daily intake
BMI	Body mass index
CPIR	Cephalic phase insulin response
EFSA	European Food Safety Authority
FDA	Food and Drug Administration
GLP-1	Glucagon-like peptide 1
GRAS	Generally recognized as safe
HbA1c	Hemoglobin A1C
HDL-c	High-density lipoprotein cholesterol
IR	Insulin resistance
JECFA	Joint FAO/WHO Expert Committee on Food Additives
LDL-c	Low-density lipoprotein cholesterol
OGTT	Oral glucose tolerance test
ND	No statistical differences detected
NGO	Non-governmental organization
NNS(s)	Non-nutritive sweetener(s)
PYY	Peptide YY
RCT(s)	Randomized controlled trial(s)
Reb	Rebaudioside
SCFAs	Short-chain fatty acids
TC	Total cholesterol
TG	Triglyceride
VLDL-c	Very-low-density lipoprotein-cholesterol
WHO-NUGAG	World Health Organization Nutrition Guidance Expert Advisory Committee
