# Supplementary material for: Stevia Rebaudiosides Usage as a Sugar Reduction Tool: A Narrative Review of Their Metabolic, Gut Microbiome and Weight Management Effects in Human Clinical Studies
Source: Nutrients. 2026 Jun 20;18(12):2002. doi: 10.3390/nu18122002 (PMC13305536; doi:10.3390/nu18122002)
Supplement: Supplementary file 1 [file nutrients-18-02002-s001.zip › Scopus Stevia Search Terms and Filters.pdf]

Scopus Search Filters

Search: Stevia Clinical Trials

(TITLE-ABS-KEY (stevia;) AND PUBYEAR > 1999 AND PUBYEAR < 2027) AND (randomized controlled trial) AND (human study) AND (clinical study) AND (LIMIT-TO (DOCTYPE , "ar")) AND (LIMIT-TO (EXACTKEYWORD , "Controlled Study") OR LIMIT-TO (EXACTKEYWORD , "Stevia") OR LIMIT-TO (EXACTKEYWORD , "Humans") OR LIMIT-TO (EXACTKEYWORD , "Glucose Blood Level") OR LIMIT-TO (EXACTKEYWORD , "Clinical Trial") OR LIMIT-TO (EXACTKEYWORD , "Appetite") OR LIMIT-TO (EXACTKEYWORD , "Blood Pressure") OR LIMIT-TO (EXACTKEYWORD , "Glycosides") OR LIMIT-TO (EXACTKEYWORD , "Human") OR LIMIT-TO (EXACTKEYWORD , "Article") OR EXCLUDE (EXACTKEYWORD , "Rat") OR LIMIT-TO (EXACTKEYWORD , "Randomized Controlled Trial") OR EXCLUDE (EXACTKEYWORD , "Stevioside") OR EXCLUDE (EXACTKEYWORD , "Nonhuman") OR EXCLUDE (EXACTKEYWORD , "Animals") OR EXCLUDE (EXACTKEYWORD , "Animal") OR EXCLUDE (EXACTKEYWORD , "Plant Extracts") OR EXCLUDE (EXACTKEYWORD , "Mouse") OR EXCLUDE (EXACTKEYWORD , "Mice") OR EXCLUDE (EXACTKEYWORD , "Steviol") OR EXCLUDE (EXACTKEYWORD , "Animal Experiment") OR EXCLUDE (EXACTKEYWORD , "Herbal Medicine") OR EXCLUDE (EXACTKEYWORD , "Stevia Rebaudiana Extract") OR EXCLUDE (EXACTKEYWORD , "Medicinal Plant") OR EXCLUDE (EXACTKEYWORD , "In Vitro Study") OR EXCLUDE (EXACTKEYWORD , "Animal Model")) AND (LIMIT-TO (LANGUAGE , "English"))
