# Supplementary material for: Stevia Rebaudiosides Usage as a Sugar Reduction Tool: A Narrative Review of Their Metabolic, Gut Microbiome and Weight Management Effects in Human Clinical Studies
Source: Nutrients. 2026 Jun 20;18(12):2002. doi: 10.3390/nu18122002 (PMC13305536; doi:10.3390/nu18122002)
Supplement: Supplementary file 1 [file nutrients-18-02002-s001.zip › Central Cochrane Search.pdf]

Search Name: Stevia Cochrane Search 5-11-26

Last Saved: 11/05/2026 13:03:27

Comment: Cochrane Stevia Clinical Trial Search

ID	Search
#1	("Stevia"):ti,ab,kw (Word variations have been searched)
#2	("Stevia"):ti,ab,kw (Word variations have been searched)
#3	("Stevia"):ti,ab,kw (Word variations have been searched)
#4	("Stevia"):ti,ab,kw OR (steviol glycosides):ti,ab,kw (Word variations have been searched)

1: Tey SL, Salleh NB, Henry J, Forde CG. Effects of aspartame-, monk fruit-, stevia- and sucrose-sweetened beverages on postprandial glucose, insulin and energy intake. Int J Obes (Lond). 2017 Mar;41(3):450-457. doi: 10.1038/ijo.2016.225. Epub 2016 Dec 13. PMID: 27956737.

2: Suez J, Cohen Y, Valdés-Mas R, Mor U, Dori-Bachash M, Federici S, Zmora N, Leshem A, Heinemann M, Linevsky R, Zur M, Ben-Zeev Brik R, Bukimer A, Eliyahu-Miller S, Metz A, Fischbein R, Sharov O, Malitsky S, Itkin M, Stettner N, Harmelin A, Shapiro H, Stein-Thoeringer CK, Segal E, Elinav E. Personalized microbiome-driven effects of non-nutritive sweeteners on human glucose tolerance. Cell. 2022 Sep 1;185(18):3307-3328.e19. doi: 10.1016/j.cell.2022.07.016. Epub 2022 Aug 19. PMID: 35987213.

3: Gibbons C, Beaulieu K, Almiron-Roig E, Navas-Carretero S, Martínez JA, O'Hara B, O'Connor D, Nazare JA, Le Bail A, Rannou C, Hardman C, Wilton M, Kjølbæk L, Scott C, Moshoyiannis H, Raben A, Harrold JA, Halford JCG, Finlayson G. Acute and two-week effects of neotame, stevia rebaudioside M and sucrose-sweetened biscuits on postprandial appetite and endocrine response in adults with overweight/obesity-a randomised crossover trial from the SWEET consortium. EBioMedicine. 2024 Apr;102:105005. doi: 10.1016/j.ebiom.2024.105005. Epub 2024

Mar 28. PMID: 38553262; PMCID: PMC11026940.

4: Sambra V, Vicuña IA, Priken KM, Luna SL, Allendes DA, Godoy PM, Novik V, Vega CA. Acute responses of stevia and d-tagatose intake on metabolic parameters and appetite/satiety in insulin resistance. Clin Nutr ESPEN. 2022 Jun;49:217-224. doi: 10.1016/j.clnesp.2022.04.018. Epub 2022 Apr 22. PMID: 35623816.

5: Stamataki NS, Scott C, Elliott R, McKie S, Bosscher D, McLaughlin JT. Stevia Beverage Consumption prior to Lunch Reduces Appetite and Total Energy Intake without Affecting Glycemia or Attentional Bias to Food Cues: A Double-Blind Randomized Controlled Trial in Healthy Adults. J Nutr. 2020 May 1;150(5):1126-1134. doi: 10.1093/jn/nxaa038. PMID: 32125421.

6: Ng AWR, Loh KK, Gupta N, Narayanan K. A polyol-stevia blended sugar replacer exhibits low glycemic response among human subjects. Clin Nutr ESPEN. 2019 Oct;33:39-41. doi: 10.1016/j.clnesp.2019.07.014. Epub 2019 Jul 31. PMID: 31451273.

7: Higgins KA, Mattes RD. A randomized controlled trial contrasting the effects of 4 low-calorie sweeteners and sucrose on body weight in adults with overweight or obesity. Am J Clin Nutr. 2019 May 1;109(5):1288-1301. doi: 10.1093/ajcn/nqy381. PMID: 30997499.

8: Farhat G, Berset V, Moore L. Effects of Stevia Extract on Postprandial Glucose Response, Satiety and Energy Intake: A Three-Arm Crossover Trial. Nutrients. 2019 Dec 12;11(12):3036. doi: 10.3390/nu11123036. PMID: 31842388; PMCID: PMC6950708.

9: Zafrilla P, Masoodi H, Cerdá B, García-Viguera C, Villaño D. Biological

effects of stevia, sucralose and sucrose in citrus-maqui juices on overweight subjects. Food Funct. 2021 Sep 20;12(18):8535-8543. doi: 10.1039/d1fo01160j. PMID: 34323244.

10: Urrutia-Espinosa M, Concha-Fuentealba F, Fuentes-Barría H, Angarita Dávila LC, Carrasco Hernández ME, Aguilera-Eguía R, Alarcón Rivera M, López Soto OP. Efectos de D-tagatosa, estevia y sacarosa sobre el pH y la actividad bacteriana oral en estudiantes de odontología. Ensayo controlado y aleatorizado [Effects of D-tagatose, Stevia and Sucrose on pH and oral bacterial activity in dentistry students. A randomized controlled trial]. Nutr Hosp. 2024 Oct 31;41(5):1091-1097. Spanish. doi: 10.20960/nh.05253. PMID: 39037177.

11: Almiron-Roig E, Navas-Carretero S, Castelnuovo G, Kjølbæk L, Romo-Hualde A, Normand M, Maloney N, Hardman CA, Hodgkins CE, Moshoyiannis H, Finlayson G, Scott C, Raats MM, Harrold JA, Raben A, Halford JCG, Martínez JA. Impact of acute consumption of beverages containing plant-based or alternative sweetener blends on postprandial appetite, food intake, metabolism, and gastro-intestinal symptoms: Results of the SWEET beverages trial. Appetite. 2023 May 1;184:106515. doi: 10.1016/j.appet.2023.106515. Epub 2023 Feb 26. PMID: 36849009.

12: Stamataki NS, Crooks B, Ahmed A, McLaughlin JT. Effects of the Daily Consumption of Stevia on Glucose Homeostasis, Body Weight, and Energy Intake: A Randomised Open-Label 12-Week Trial in Healthy Adults. Nutrients. 2020 Oct 6;12(10):3049. doi: 10.3390/nu12103049. PMID: 33036155; PMCID: PMC7600789.

13: Ahmad J, Khan I, Johnson SK, Alam I, Din ZU. Effect of Incorporating Stevia and Moringa in Cookies on Postprandial Glycemia, Appetite, Palatability, and Gastrointestinal Well-Being. J Am Coll Nutr. 2018 Feb;37(2):133-139. doi: 10.1080/07315724.2017.1372821. Epub 2017 Dec 22. PMID: 29272206.

14: Stamataki NS, Mckie S, Scott C, Bosscher D, Elliott R, McLaughlin JT. Mapping the Homeostatic and Hedonic Brain Responses to Stevia Compared to Caloric Sweeteners and Water: A Double-Blind Randomised Controlled Crossover Trial in Healthy Adults. Nutrients. 2022 Oct 7;14(19):4172. doi: 10.3390/nu14194172. PMID: 36235824; PMCID: PMC9570671.

15: Pallepati A, Yavagal P, Veeresh DJ. Effect of Consuming Tea with Stevia on Salivary pH - An In Vivo Randomised Controlled Trial. Oral Health Prev Dent. 2017;15(4):315-319. doi: 10.3290/j.ohpd.a38572. PMID: 28681047.

16: Kwok D, Scott C, Strom N, Au-Yeung F, Lam C, Chakrabarti A, Hutton T, Wolever TM. Comparison of a Daily Steviol Glycoside Beverage compared with a Sucrose Beverage for Four Weeks on Gut Microbiome in Healthy Adults. J Nutr. 2024 Apr;154(4):1298-1308. doi: 10.1016/j.tjnut.2024.01.032. Epub 2024 Feb 24. PMID: 38408729.

17: Angarita Dávila L, Durán Agüero S, Apaicio D, Parra K, Uzcátegui M, Céspedes V, Reina Villasmil N, López Miranda J. Rol de la estevia y L-carnitina sobre el impacto glicémico de un suplemento nutricional en adultos [Role of the stevia and L-carnitine of a nutritional supplement on glycemic impact in adults]. Nutr Hosp. 2017 Nov 16;34(5):1455-1462. Spanish. doi: 10.20960/nh.1153. PMID: 29280664.

18: Brambilla E, Cagetti MG, Ionescu A, Campus G, Lingström P. An in vitro and in vivo comparison of the effect of Stevia rebaudiana extracts on different caries-related variables: a randomized controlled trial pilot study. Caries Res. 2014;48(1):19-23. doi: 10.1159/000351650. Epub 2013 Nov 6. PMID: 24216624.

19: Goodman S, Vanderlee L, Jones A, White C MSc, Hammond D. Perceived Healthiness of Sweeteners among Young Adults in Canada. Can J Diet Pract Res. 2021 Jun 1;82(2):90-94. doi: 10.3148/cjdpr-2020-030. Epub 2020 Dec 15. PMID: 33320777.

20: Galicia-Ayala C, Klünder-Klünder M, Vilchis-Ordoñez A, López-Martínez B, Miranda-Lora AL. Enteroendocrine hormonal response after the ingestion of cola beverages with sucrose and non-nutritive sweeteners in healthy adults: A randomized crossover trial. Nutrition. 2026 Jan;141:112938. doi: 10.1016/j.nut.2025.112938. Epub 2025 Aug 25. PMID: 41016269.

21: Tey SL, Salleh NB, Henry CJ, Forde CG. Effects of non-nutritive (artificial vs natural) sweeteners on 24-h glucose profiles. Eur J Clin Nutr. 2017 Sep;71(9):1129-1132. doi: 10.1038/ejcn.2017.37. Epub 2017 Apr 5. PMID: 28378852.

22: Mooradian AD. In search for an alternative to sugar to reduce obesity. Int J Vitam Nutr Res. 2019 Sep;89(3-4):113-117. doi: 10.1024/0300-9831/a000531. Epub 2019 Feb 12. PMID: 30747604.

23: Large T, Williams J Jr, Asplin JR, Krambeck A. Using Low-Calorie Orange Juice as a Dietary Alternative to Alkali Therapy. J Endourol. 2020 Oct;34(10):1082-1087. doi: 10.1089/end.2020.0031. Epub 2020 Aug 4. PMID: 32363926.

24: Mohire NC, Yadav AV. Chitosan-based polyherbal toothpaste: as novel oral hygiene product. Indian J Dent Res. 2010 Jul-Sep;21(3):380-4. doi: 10.4103/0970-9290.70808. PMID: 20930349.

25: Gregersen S, Jeppesen PB, Holst JJ, Hermansen K. Antihyperglycemic effects

of stevioside in type 2 diabetic subjects. Metabolism. 2004 Jan;53(1):73-6. doi: 10.1016/j.metabol.2003.07.013. PMID: 14681845.

26: Agulló V, Domínguez-Perles R, García-Viguera C. Sweetener influences plasma concentration of flavonoids in humans after an acute intake of a new (poly)phenol-rich beverage. Nutr Metab Cardiovasc Dis. 2021 Mar 10;31(3):930-938. doi: 10.1016/j.numecd.2020.11.016. Epub 2020 Nov 24. PMID: 33546941.

27: Lian K, Hammarström D, Hamarsland H, Mølmen KS, Moen SC, Ellefsen S. Glucose ingestion before and after resistance training sessions does not augment ribosome biogenesis in healthy moderately trained young adults. Eur J Appl Physiol. 2024 Aug;124(8):2329-2342. doi: 10.1007/s00421-024-05446-x. Epub 2024 Mar 8. PMID: 38459192; PMCID: PMC11322406.

28: Ferri LA, Alves-Do-Prado W, Yamada SS, Gazola S, Batista MR, Bazotte RB. Investigation of the antihypertensive effect of oral crude stevioside in patients with mild essential hypertension. Phytother Res. 2006 Sep;20(9):732-6. doi: 10.1002/ptr.1944. PMID: 16775813.

29: Hengist A, Davies RG, Rogers PJ, Brunstrom JM, van Loon LJC, Walhin JP, Thompson D, Koumanov F, Betts JA, Gonzalez JT. Restricting sugar or carbohydrate intake does not impact physical activity level or energy intake over 24 h despite changes in substrate use: a randomised crossover study in healthy men and women. Eur J Nutr. 2023 Mar;62(2):921-940. doi: 10.1007/s00394-022-03048-x. Epub 2022 Nov 3. PMID: 36326863; PMCID: PMC9941259.

30: Barriocanal LA, Palacios M, Benitez G, Benitez S, Jimenez JT, Jimenez N, Rojas V. Apparent lack of pharmacological effect of steviol glycosides used as

sweeteners in humans. A pilot study of repeated exposures in some normotensive and hypotensive individuals and in Type 1 and Type 2 diabetics. Regul Toxicol Pharmacol. 2008 Jun;51(1):37-41. doi: 10.1016/j.yrtph.2008.02.006. Epub 2008 Mar 5. PMID: 18397817.

31: Maki KC, Curry LL, Carakostas MC, Tarka SM, Reeves MS, Farmer MV, McKenney JM, Toth PD, Schwartz SL, Lubin BC, Dicklin MR, Boileau AC, Bisognano JD. The hemodynamic effects of rebaudioside A in healthy adults with normal and low-normal blood pressure. Food Chem Toxicol. 2008 Jul;46 Suppl 7:S40-6. doi: 10.1016/j.fct.2008.04.040. Epub 2008 May 16. PMID: 18555574.

32: Samant SS, Wilkes K, Odek Z, Seo HS. Tea-induced calmness: Sugar-sweetened tea calms consumers exposed to acute stressor. Sci Rep. 2016 Nov 16;6:36537. doi: 10.1038/srep36537. PMID: 27848976; PMCID: PMC5111075.

33: Rogers PJ, Ferriday D, Irani B, Hei Hoi JK, England CY, Bajwa KK, Gough T. Sweet satiation: Acute effects of consumption of sweet drinks on appetite for and intake of sweet and non-sweet foods. Appetite. 2020 Jun 1;149:104631. doi: 10.1016/j.appet.2020.104631. Epub 2020 Feb 11. PMID: 32057842.

34: Clouard C, Val-Laillet D. Impact of sensory feed additives on feed intake, feed preferences, and growth of female piglets during the early postweaning period. J Anim Sci. 2014 May;92(5):2133-40. doi: 10.2527/jas.2013-6809. Epub 2014 Mar 25. PMID: 24668952.

35: Chan P, Tomlinson B, Chen YJ, Liu JC, Hsieh MH, Cheng JT. A double-blind placebo-controlled study of the effectiveness and tolerability of oral stevioside in human hypertension. Br J Clin Pharmacol. 2000 Sep;50(3):215-20. doi: 10.1046/j.1365-2125.2000.00260.x. PMID: 10971305; PMCID: PMC2014988.

36: Taghizadeh M, Asemi Z. Effects of synbiotic food consumption on glycemic status and serum hs-CRP in pregnant women: a randomized controlled clinical trial. Hormones (Athens). 2014 Jul-Sep;13(3):398-406. doi: 10.14310/horm.2002.1489. PMID: 25079465.

37: Asemi Z, Khorrami-Rad A, Alizadeh SA, Shakeri H, Esmaillzadeh A. Effects of synbiotic food consumption on metabolic status of diabetic patients: a double-blind randomized cross-over controlled clinical trial. Clin Nutr. 2014 Apr;33(2):198-203. doi: 10.1016/j.clnu.2013.05.015. Epub 2013 Jun 7. Retraction in: Clin Nutr. 2025 Oct;53:284. doi: 10.1016/j.clnu.2025.08.016. PMID: 23786900.

38: Agulló V, García-Viguera C, Domínguez-Perles R. Beverages Based on Second Quality Citrus Fruits and Maqui Berry, a Source of Bioactive (Poly)phenols: Sorting Out Urine Metabolites upon a Longitudinal Study. Nutrients. 2021 Jan 22;13(2):312. doi: 10.3390/nu13020312. PMID: 33499139; PMCID: PMC7910882.

39: Hsieh MH, Chan P, Sue YM, Liu JC, Liang TH, Huang TY, Tomlinson B, Chow MS, Kao PF, Chen YJ. Efficacy and tolerability of oral stevioside in patients with mild essential hypertension: a two-year, randomized, placebo-controlled study. Clin Ther. 2003 Nov;25(11):2797-808. doi: 10.1016/s0149-2918(03)80334-x. PMID: 14693305.
